# Gal9/Tim-3 expression level is higher in AML patients who fail chemotherapy

**DOI:** 10.1186/s40425-019-0611-3

**Published:** 2019-07-10

**Authors:** Paola Dama, Marshall Tang, Noreen Fulton, Justin Kline, Hongtao Liu

**Affiliations:** University of Chicago Medicine Hematology/Oncology Section, 5841 S Maryland Ave, Chicago, IL 60637 USA

**Keywords:** Acute myeloid leukemia, Chemotherapy, Immune checkpoint, Treatment failure, T-cell, Exhaustion, Beyond PD-1, TIM-3

## Abstract

Immune checkpoint pathways active in Acute Myeloid Leukemia (AML) patients, especially during the course of remission induction chemotherapy, have not been well studied. Although dominant in mediating T cell dysfunction in cancer, it is now well-accepted that interruption of PD-1/PD-L1 axes alone does not always completely restore T cell function, indicating the involvement of additional negative regulatory pathways, such as TIM-3/Gal-9, in promoting T cell exhaustion.

Here, we characterized these pathways in AML patients enrolled in a phase I dose escalation trial that combined Selinexor, a Selective Inhibitor of Nuclear Export (SINE), with high-dose cytarabine (HiDAC) and mitoxantrone (Mito) (NCT02573363) as induction therapy.

To monitor changes in expression of immune checkpoint receptors, multi-parameter flow cytometry was performed on peripheral blood and bone marrow biopsy specimens at diagnosis and following induction therapy in 26 AML patients. Expression of CD47, PD-L1, PD-L2 and Gal9 was assessed on CD34^+^ AML blasts, as well as on CD34^−^ cell populations. In parallel, we evaluated expression of inhibitory (PD1, CTLA4, LAG3, TIM-3) and stimulatory (CD28, ICOS, CD137, OX40, CD40L, HLA-DR) co-receptors on CD4^+^ and CD8^+^ T cell subsets.

Compared to baseline, the frequency of Gal9^+^ CD34^−^ cells was significantly higher in patients with treatment failure (TF) than in those in complete remission (CR), and this finding correlated with increased TIM-3 expression on marrow-resident T cells in TF patients. Moreover, when we measured the expression level of PD-1 and TIM-3 in bone marrow samples compared to peripheral blood, TIM-3 was significantly higher in BM specimens.

Our results suggest that targeting the Gal9/Tim-3 axis could be effective in combination with induction chemotherapy to increase the likelihood of complete remission in AML patients.

**Electronic supplementary material:**

The online version of this article (10.1186/s40425-019-0611-3) contains supplementary material, which is available to authorized users.

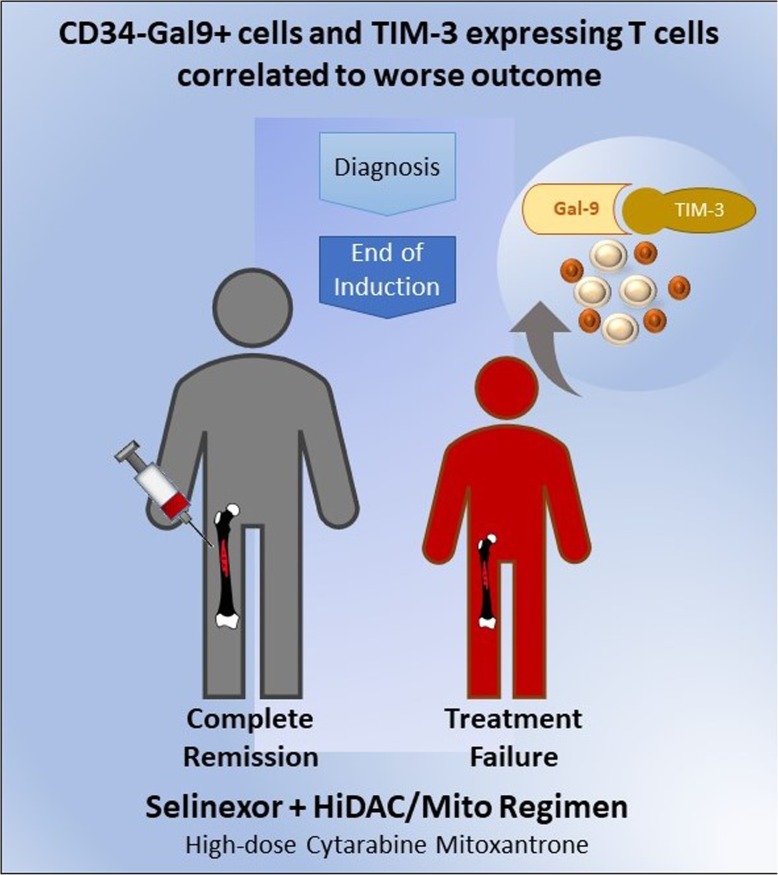

## Introduction

Acute Myeloid Leukemia (AML) is characterized by a poor prognosis, even in patients who achieve a complete remission to initial therapy. Indeed, leukemia cells exploit a variety of mechanisms to evade T-cell-mediated immunity, leading to disease progression and relapse [1–4].

Among them, activation of immune checkpoint pathways in AML may interfere with effective T-cell antitumor immunity. PD-1/PD-L1 interactions are associated with immune evasion in pre-clinical leukemia models, as we and others have previously demonstrated [5, 6]. It was also reported that overexpression of PD-1 on stroma/non-blast compartment and its ligands (PD-L1 and PD-L2) on CD34^+^ leukemia cells is associated with more aggressive leukemia and progression from Myelodysplastic syndromes (MDS) to AML or AML relapse [7, 8]. Importantly patients resistant to epigenetic therapy had relatively higher increments in expression of these genes compared with patients who achieved response [8]. Clinical studies of PD-1 blockade are currently ongoing in patients with AML and MDS [9].

Although dominant in mediating T cell dysfunction in cancer, it is now well accepted that interruption of PD-1/PD-L1 axes alone does not completely restore T cell function in some patients, indicating the involvement of additional negative regulatory pathways, such as TIM-3/Gal-9, in promoting T cell exhaustion [10, 11]. T cell immunoglobulin and mucin domain 3 (TIM-3) is expressed on Th1, Th17, CD8+ T cells–cells of myeloid lineages [12] in mice. The inhibitory role of TIM-3 in T cell-mediated immune responses is produced by the binding of Galectin-9, one of its ligands, to the carbohydrate motif on TIM-3 inducing Th1 and Th17 cell death [13, 14].

Combinational targeting of these pathways has recently been proposed in hematological malignancies. Administration of TIM-3 and PD-1 mAbs synergistically control tumor growth [10].

Recently, we conducted a prospective phase I clinical trial of Selinexor, a Selective Inhibitor of Nuclear Export (SINE), combined with High-Dose Cytarabine Mitoxantrone (NCT02573363). HiDAC + Mito is an effective induction regimen frequently utilized for patients with high-risk AML, either de novo or relapsed/refractory. The clinical outcomes of patients treated in this study have been recently published [15].

Selinexor (KPT-330) is an exportin 1 (XPO1) inhibitor. XPO1 is a nuclear export receptor involved in a cytoplasmatic translocation of most major tumor suppressor proteins (TSP) and growth regulatory proteins (GRP), including p53, p21, p73, FOXO1, β-catenin and NPM1 [16]. Kojima et al. have demonstrated that increased expression of XPO1 have been independently associated with a worse prognosis in adults with AML. Overexpressed levels of XPO1 lead to enhanced transport of TSP/GRP to the cytoplasm thus, forcing nuclear retention of these proteins is a rational therapeutic strategy of selinexor use in AML [17, 18].

In this short report, we first aimed to characterize the expression of such immune checkpoint molecules, both on CD34^+^ AML cells and on CD34^−^ bone marrow cells and on blood and bone marrow (BM) resident T cells during the treatment course. The rationale and sustainability of incorporating checkpoint blockade once the patients achieve remission as a means of providing immune-mediated protection from relapse is a challenging question of recent years.

Furthermore, in order to better understand the possible mechanism in immune response of the resistance to chemotherapy, patients enrolled in this study were divided in two groups - those in complete remission (CR) and those that experienced resistant disease, relapse, or death prior to, or as the result of treatment failure (TF). The comparison between them was employed at diagnosis, end of induction and at the point of primary induction failure.

The characterization and comparison of immune checkpoint ligands and receptors in the bone marrow at time of diagnosis and end of induction, allowed us to monitor the changes and to identify predictive or prognostic biomarkers to guide future immunotherapy in AML.

## Methods

### Treatment regimen and sample collection

Table 1 represents the characteristics of 26 patients divided in two groups enrolled to a phase I dose escalation trial that combined increasing doses of Selinexor (SINE) with age-adjusted HiDAC/Mito (NCT02573363) at time of diagnosis. Patients that experienced induction failure were taken off of protocol therapy due to death or documented induction failure. HiDAC (3 g/m2,or 2 g/m2 if > 70 years, intravenously over 4 h) followed immediately by Mito (30 mg/m2, or 20 mg/m2 if > 70 years, intravenously over 1 h) was administered on days 1 and 5. Selinexor was given orally on days 2, 4, 9, and 11. Initial Selinexor dose was 60 mg (~ 35 mg/m2 for an average adult) followed by dose escalation to a target level of 80 mg (~ 50 mg/m2). Bone marrow (BM) and blood samples were collected at the time of diagnosis and at the end of induction/treatment (days-range 19–56). (Additional file 1: Figure S1-A and S1-B).

**Table 1:** Characteristic of the patients

	Disease Response	
	Complete Remission CR	Treatment Failure TF
Patient Characteristics	Number (%)	Number (%)
Total patients enrolled	16	10
Female	6 (37%)	8 (80%)
Median Age (years, range)	61 (35–75)	62 (38–74)
Disease State on enrollment*		
Untreated AML	12 (80%)	2 (22%)
Relapse/refractory AML	3 (20%)	7 (78%)
Initial AML diagnosis*		
De Novo AML	8 (53%)	5 (55%)
Secondary AML after MDS	7 (47%)	4 (45%)
Acquired Mutation Status*		
FLT3	3 (20%) NMP1 mutated	2 (22%) NMP1 mutated
CEPBA	1 (6%)	2 (22%)
NMP1	5 (34%)	1 (11%)
Blast (CD34+)		
Median	9.90%	48.90%
Range	4.5–89.7	7.9–76.6

* CR pts. = 15 TF* pts. = 9

### Flow cytometry

At time of diagnosis, multi-parameter flow cytometry was performed on blood and bone marrow (BM) aspirates. Expression of CD47 PerCP-Cy5.5 (BioLegend Clone CC2C6), PD-L1 BV-421(BioLegend Clone 29E.2A3), PD-L2 Pe (BioLegend Clone 24F.10C12) and Gal-9 APC (BioLegend Clone 9 M1–3) was assessed on CD34^+^ FITC (BioLegend Clone 581) AML blasts and on stroma/non-blast compartment CD34^−^ cell populations. In parallel, we evaluated expression of inhibitory PD1 Pe (BioLegend Clone EH12.2H7), CTLA4 APC (BioLegend Clone L3D10), LAG3 Pe-Cy7(eBioscience Clone 3DS223H), TIM3 APC (Cy-7 BioLegend clone F38-2E2) and stimulatory CD28 APC (BioLegend Clone CD28.2), ICOS APC-Cy7 (BDBiosciences clone C398.4A), CD137 APC (BioLegend Clone 4B4–1), OX40 APC (BioLegend Clone Ber-ACT35, CD40L Pe-Cy7 (BioLegend Clone 24–31), HLA-DR APC (BioLegend clone L243) co-receptors on CD4^+^ (PerCPCy5.5 BioLegend Clone SK3) and CD8^+^ (FITC BioLegend Clone HIT8a) T cell subsets. A Fluorescence Minus One (FMO) controls were used to determine the median fluorescence intensity (MFI) and frequency among the parent population of each costimulatory and coinhibitory molecule. (Additional file 1: Figure S2A) Flow cytometry was performed on LSR Fortessa or LSRII cytometers. Data were analyzed with FlowJo-10 software.

### Statistics

The Mann Whitney Test, Spearman’s rank correlation and Runs Test analysis were applied to compare the difference in two group of patients - those in complete remission (CR) defined with an absolute neutrophil count (ANC) > 1.0 × 109/L, platelet count > 100 × 109/L, and bone marrow blasts < 5% [19, 20] and those that experienced resistant disease, relapse, or death prior or as results of treatment failure (TF). For all analyses, *P*-values <0.05 were considered statistically significant.

## Results and discussion

In this analysis, we aimed to characterize dynamic changes in expression of immune checkpoint pathways on AML cells and T cells resident in bone marrow environment and peripheral blood prior to and after induction chemotherapy.

Specimens from patients with high-risk AML enrolled in a prospective clinical trial that combined Selinexor with HiDAC+Mito (NCT02573363) were employed to address the question of the incorporating checkpoint blockade in combination with chemotherapy as a means of immune-mediated protection, even for those patients that achieve remission.

To monitor changes in expression profiles of immune checkpoint receptors and ligands, multi-parameter flow cytometry was performed on bone marrow (BM) aspirates and peripheral blood from 26 patients with AML at the time of diagnosis and at the end of induction chemotherapy. Patients were divided into 2 cohorts - those who achieved CR (*n* = 16), and those who experienced TF (*n* = 10) (Table 1). Additional file 1: Figure S1A shows the diagram of the strategy of our study and sample collection. Kaplan-Meier plot (Additional file 1: Figure S1B) depicts patient survival from the time of diagnosis to CR or TF populations. The median of days elapsed from diagnosis was 346 and 176 for CR and TF respectively, and Hazard Ratio (Mantel-Haenszel) TF/CR was 1.7; Mantel-Cox test was not significant. The shadow in the chart indicates the timewise of samples collection and analysis.

**Fig. 1:**
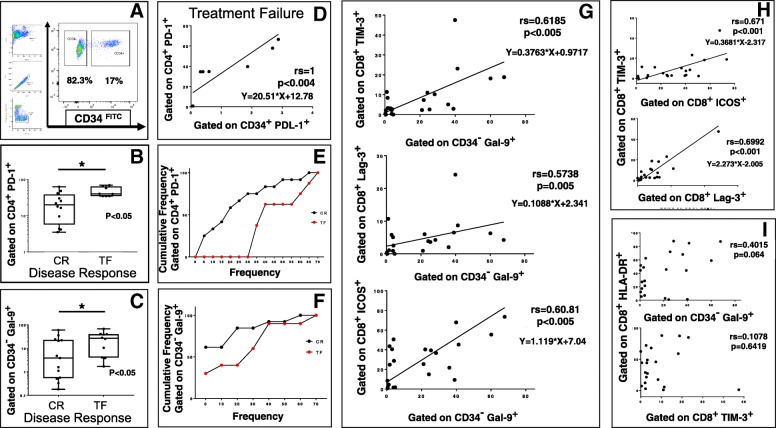
The association between Gal9 and TIM-3 as prognostic marker for Selinexor + HiDAC Mito regimen. At time of diagnosis, multi-parameter flow-cytometry was performed on bone marrow (BM) aspirates from 26 patients. A FITC conjugated anti-CD34 antibody was used to analyze frequencies of CD34+ AML cells and the remaining CD34− cell populations. (**a**) Patients were divided into 2 cohorts - those who achieved CR (n = 16), and those who experienced TF (n = 10). The comparison of frequency of CD4+ PD-1+ T cells and CD34−Gal-9+ in these two groups are shown. (**b**-**c**) Bars represent medians. Percentage of cumulative frequencies are displayed in (**e**) and (**f**). We calculated the Spearman correlation coefficients to describe associations between CD4+PD-1+ T cells and CD34+PDL-1+ AML cells in TF patients (**d**); same for CD34-Gal-9+ between inhibitory and activator markers TIM-3, ICOS, Lag3 on CD8+ cells, at time of diagnosis (**g**). Lastly, between CD8+TIM-3+ and ICOS and Lag-3. (**h**) A linear regression according to the Deming procedure and deviation for linearity (Runs Test) was additionally computed. Gal-9 and TIM-3 vs HLA-DR was used as negative control.(**i**)

An anti-CD34 antibody was used to analyze frequencies of CD34^+^ AML cells and the remaining CD34^−^ cell populations and to evaluate expression of costimulatory and coinhibitory ligands on the respective cell populations (Fig. 1a).

Not surprisingly, there was a statistical trend toward higher frequencies of CD34^+^ cells in TF patients compared to CR patients in diagnostic BM specimens (48.9%, range: 7.9–76.6% versus 9.9%, range: 4.5–89.7%; *p* = 0.07), although there was a high degree of inter-patient variability. (Additional file 1: Figure S3). In this regard the report of Kanda et al., where they reviewed 22 studies, encompassing 2483 patients, the investigation on clinical significance of CD34 expression in AML as an adverse prognostic marker had contradictory results [5].

Yang et al. in 2014 demonstrated that PD-1 signaling may be involved in MDS pathogenesis and resistance mechanisms to hypomethylating agents. In AML and MDS bone marrow biopsies blasts were positive for PD-L1 whereas stroma/non-blast cellular compartment was positive for PD-1 suggesting that PD-1 ligand expressed on tumor cells may act through PD-1 stroma within the tumor microenvironment [8].

At the time of diagnosis, the frequency of PD-1^+^CD4^+^ T cells was higher in TF patients compared to CR patients (Fig. 1b and e) as well as PD-1 + CD8+ T cells even if it is not significant in the latter case (Additional file 1: Figure S4A).

In order to understand this finding, we calculated the Spearman correlation coefficients to describe the association between PD-L1 and PD-1 expression in these two populations. As shown in Fig. 1d, there was a strong correlation between CD4^+^PD1^+^ T cells and CD34^+^PD-L1^+^ AML cells only in TF patients (rs = 1; *p* < 0.0004) comparing to CR patients (rs = 0.43; *p* = 0.2, data not shown). The same trend is express PD-1 + CD8+ T cells. (Additional file 1: Figure S4B). However, the expression of PD-L1 on CD34+ AML cells was lower in TF patients than in those who achieved CR (Additional file 1: Figure S4C).

Interestingly, the percentage of Gal-9^+^CD34^−^ cells was significantly higher in TF patients compared to CR patients, with a median percentage of 26.9% (range: 1.7–67.8%) versus a median of 3.9% (range: 0.18–60.1%; *p* < 0.05, Mann Whitney Test) (Fig. 1c and f).

Increased Gal-9 expression on CD34^−^ cells was correlated with TIM-3, Lag3 and ICOS expression on bone marrow resident T cells at the time of diagnosis. A linear regression according to the Deming procedure and deviation for linearity (Runs Test) was additionally calculated and was not significant (Fig. 1g). Figure 1h illustrates the positive correlation between TIM-3 vs Lag3 and ICOS on CD8^+^ cells. Lastly, HLA-DR that is commonly expressed in AML, was not significantly correlated with Gal-9 and TIM-3 expression. (Fig. 1i).

**Fig. 2:**
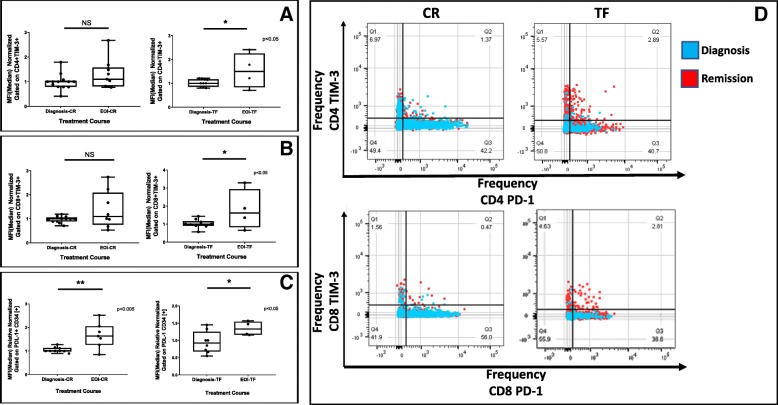
The increase of TIM-3 expression was higher in TF compare to CR patients. Median Fluorescence Intensity (MFI) was calculated by FlowJo-10 software and relative normalized to comparing TIM-3 expressing T cells (CD4+ and CD8+) and PDL-1+ CD34+ cells in CR and TF patients at time of diagnosis and end of induction (**a**-**b**). Comparison of PDL-1 expression on CD34+ AML cells in CR and TF patients. (**c**) The colored overlay dot plots show the co-expression of TIM-3 and PD-1 on CD4+ and CD8+ cells comparing expression levels of these receptors in representative patients CR (blue dot) and TF (red dot) at diagnosis vs end of induction (**d**)

We next evaluated expression of Gal-9 and TIM-3 in CD34^+^ and CD34^−^ bone marrow cells, and on bone marrow resident T cells, respectively, after induction therapy in CR and TF patients. Frequencies of TIM-3^+^CD4^+^ and TIM-3^+^CD8^+^ T-cells were heterogenous within the groups (Additional file 1: Figure S5-A and S5-B). As shown in Fig. 2a and b, we observed modestly increased expression of the normalized median fluorescence intensity (MFI) of TIM-3 on CD4^+^ and CD8^+^ T cells in CR patients compared to TIM-3 expression on T cells in diagnostic samples. The increase in TIM-3 MFI on CD4^+^ and CD8^+^ T cells was > 50% in TF cases at remission in comparison with the counterpart patients at diagnosis (Fig. 2a and b). PD-L1 expression on CD34^+^ AML cells, conversely, was similar in both TF and CR patients (Fig. 2c).

In Fig. 2d, the co-expression of TIM-3 and PD-1 on CD4^+^ and CD8^+^ cells in CR and TF patients is shown, comparing expression levels of these receptors at the point of diagnosis with their expression at the time of CR or TF. The colored overlay dot plots shows that even if the trend is the same in these two cases selected as representative for CR and TF, the frequency of TIM-3 PD-1 at time of remission in TF is higher (red dot).

It has been demonstrated that NF-kB can enhance Wnt-signaling leading to the dedifferentiation of epithelial non-stem cells into tumor-initiating cells [21]. Related to this finding, Kikushige et al. in 2015 demonstrated that canonical Wnt pathway is activated in myeloid leukemia stem cells by an autocrine TIM-3/Gal-9 signaling. In their study, they identified TIM-3 as a leukemic stem cells (LSCs) specific surface marker. AML cells secrete a high amount of Gal-9 into patient’ sera, hence they describe TIM-3/Gal-9 pathway as an essential stimulatory loop for LSCs. TIM-3 signaling activates both NF-kB and β-catenin pathways. In an experiment on immune-deficient mice the reconstitution of human AML was inhibited by neutralization of Gal-9 [22]. Noteworthy are the results of Kikushige et al. that indicate the nucleus translocation of β-catenin occurs in TIM-3+ AML cells in response to the Gal-9 ligation. Our hypothesis is that Selinexor does not affect inhibitory pathways [23] but might cause in those patients that express higher level of Gal-9 the retention of β-catenin in the nucleus leading to maintenance of AML LSCs and thus the failure of treatment.

Lastly, a previous study has demonstrated a significant difference in T cell immune response between bone marrow and peripheral blood. The group of Hong Zheng [24] reported an increased proportion of CD8 PD-1 T cells within bone marrow in a cohort of 22 patients with newly diagnosed AML. This study highlighted the importance of evaluating bone marrow samples in order to understand the complex microenvironment of the BM, which is known to be a key player in the pathogenesis of the disease. AML is derived from myeloid hematopoietic progenitors characterized by the rapid growth of abnormal cells in bone marrow before mobilizing to peripheral blood. Hence the importance in understanding the BM microenvironment in development and progression of this disease [25]. As shown in Additional file 1: Figure S6A and S6B, the comparison of bone marrow and peripheral blood samples of our patients at the time of diagnosis showed a significantly higher TIM-3 expression in both subsets of CD4 and CD8 populations. Interestingly, PD-1 expression level was not different in these two compartments in contradiction to the study of Jia et al [24].

## Conclusions

The current debate to novel therapeutic approaches that can challenge development of resistance to the treatment or relapse experienced by AML patients is direct toward the evidence of the BM microenvironment as a niche for AML [25]. In this context, despite the limit of our small group of patients, our findings suggest that the Gal9/TIM3 pathway may play a role in patients in remission by subverting ongoing immune surveillance, and suggests that T cells in AML patients, even those who achieve CR to therapy, are likely exhausted or dysfunctional.

In conclusion, the high expression of Gal-9 at diagnosis and the increased expression of TIM-3 at remission in TF patients, provides a rationale for incorporating antibodies against the Gal9/TIM3 pathway during and/or following remission induction therapy for AML. A larger cohort analysis and more mechanistic study will be needed to expand and confirm these results.

## Additional file

Additional file 1: **Figure S1.** Strategy of the previous study and samples collection. **Figure S2.** Sequential gating to identify PD-1. **Figure S3.** Statistical trend toward higher frequencies of CD34+ cells in TF patients. **Figure S4.** PD-1/PDL-1 axes at the time of diagnosis as a prognostic factor. **Figure S5.** TIM-3 expression in TF and CR patients at diagnosis and at the end of induction. **Figure S6.** TIM-3 expression in BM compartment is significant higher in the both subsets of CD4 and CD8 populations. (PDF 1480 kb)

## Abbreviations

AML: Acute Myeloid Leukemia
BM: Bone Marrow
CR: Complete Remission
FMO: Fluorescence Minus One
HiDAC: High-Dose Cytarabine
LSCs: Leukemic Stem Cells
MFI: Median Fluorescence Intensity
MITO: Mitoxantrone
PBMC: Peripheral Blood Mononuclear Cell
SINE: Selective Inhibitor of Nuclear Export
TF: Treatment Failure
