# Supplementary material for: Gal9/Tim-3 expression level is higher in AML patients who fail chemotherapy
Source: J Immunother Cancer. 2019 Jul 10;7:175. doi: 10.1186/s40425-019-0611-3 (PMC6621946; doi:10.1186/s40425-019-0611-3)
Supplement: Supplementary file 1 — Figure S1. Strategy of the previous study and samples collection. Figure S2. Sequential gating to identify PD-1. Figure S3. Statistical trend toward higher frequencies of CD34+ cells in TF patients. Figure S4. PD-1/PDL-1 axes at the time of diagnosis as a prognostic factor. Figure S5. TIM-3 expression in TF and CR patients at diagnosis and at the end of induction. Figure S6. TIM-3 expression in BM compartment is significant higher in the both subsets of CD4 and CD8 populations. (PDF 1480 kb) [file 40425_2019_611_MOESM1_ESM.pdf]

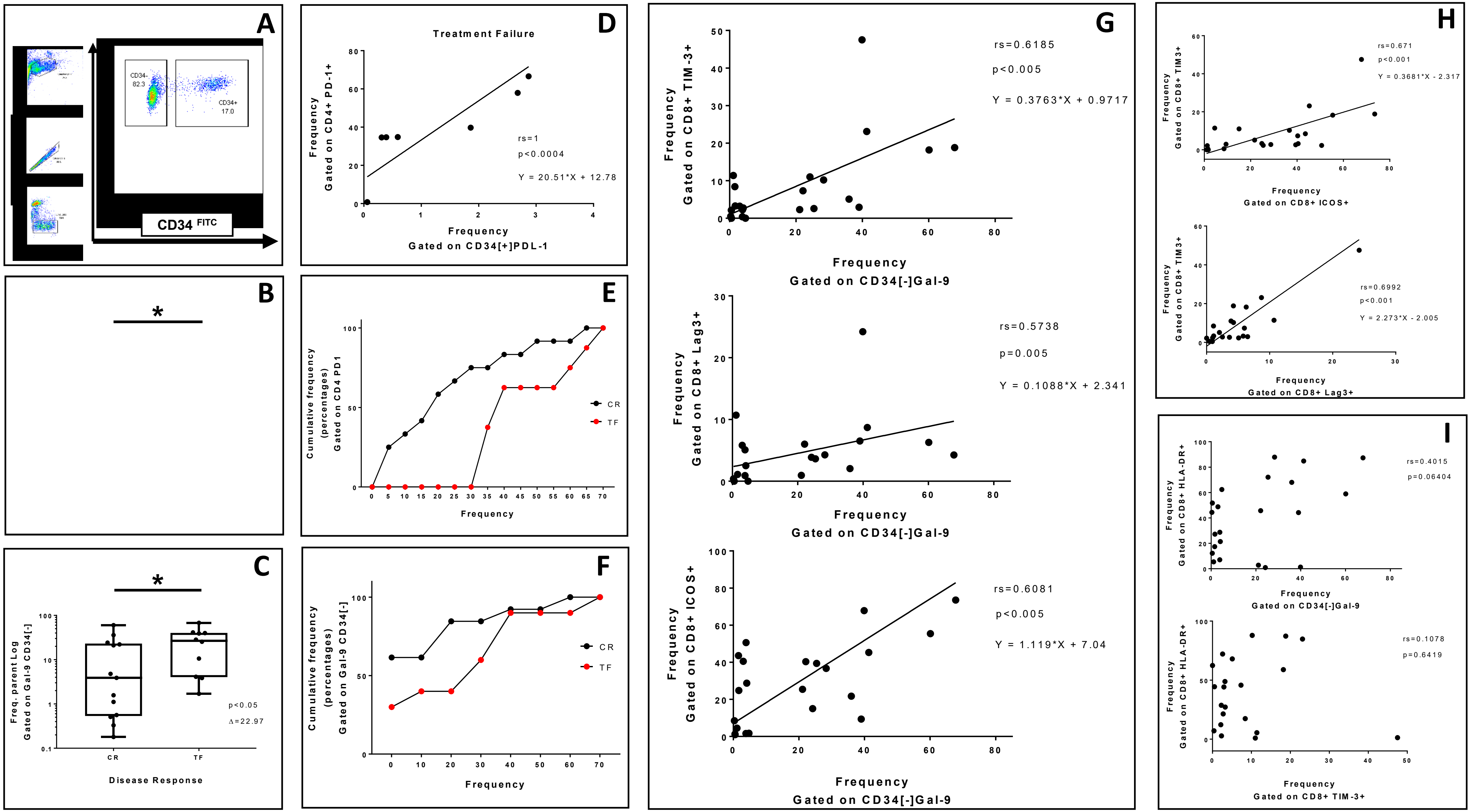
A
CD34-
82.3
CD34+
17.0
CD34 FITC
B
*
C
*
Freq. parent Log
Gated on Gal-9 CD34[-]
CR
TF
Disease Response
p<0.05
Δ=22.97
D
Treatment Failure
Frequency
Gated on CD4+ PD-1+
rs=1
p<0.0004
Y = 20.51*X + 12.78
Frequency
Gated on CD34[+]PDL-1
E
Cumulative frequency
(percentages)
Gated on CD4 PD1
CR
TF
Frequency
F
Cumulative frequency
(percentages)
Gated on Gal-9 CD34[-]
CR
TF
Frequency
G
Frequency
Gated on CD8+ TIM-3+
rs=0.6185
p<0.005
Y = 0.3763*X + 0.9717
Frequency
Gated on CD34[-]Gal-9
Frequency
Gated on CD8+ Lag3+
rs=0.5738
p=0.005
Y = 0.1088*X + 2.341
Frequency
Gated on CD34[-]Gal-9
Frequency
Gated on CD8+ ICOS+
rs=0.6081
p<0.005
Y = 1.119*X + 7.04
Frequency
Gated on CD34[-]Gal-9
H
Frequency
Gated on CD8+ TIM3+
rs=0.671
p<0.001
Y = 0.3681*X - 2.317
Frequency
Gated on CD8+ ICOS+
Frequency
Gated on CD8+ TIM3+
rs=0.6992
p<0.001
Y = 2.273*X - 2.005
Frequency
Gated on CD8+ Lag3+
I
Frequency
Gated on CD8+ HLA-DR+
rs=0.4015
p=0.06404
Frequency
Gated on CD34[-]Gal-9
Frequency
Gated on CD8+ HLA-DR+
rs=0.1078
p=0.6419
Frequency
Gated on CD8+ TIM-3+

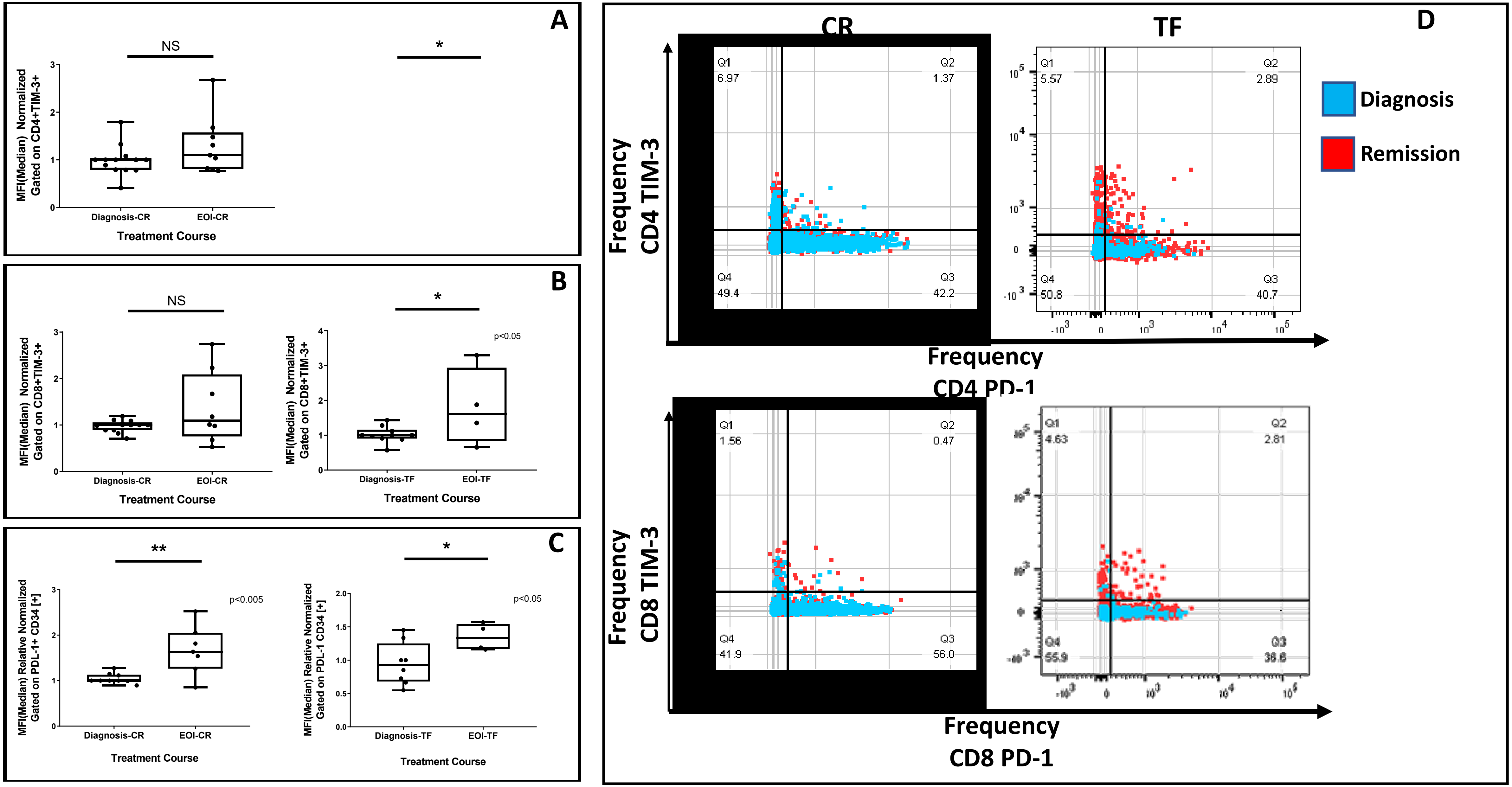
A
NS
*
MFI(Median) Normalized Gated on CD4+TIM-3+
Diagnosis-CR
EOI-CR
Treatment Course
B
NS
*
MFI(Median) Normalized Gated on CD8+TIM-3+
Diagnosis-CR
EOI-CR
Treatment Course
p<0.05
Diagnosis-TF
EOI-TF
Treatment Course
C
**
*
MFI(Median) Relative Normalized Gated on PDL-1+ CD34 [+]
p<0.005
Diagnosis-CR
EOI-CR
Treatment Course
MFI(Median) Relative Normalized Gated on PDL-1 CD34 [+]
p<0.05
Diagnosis-TF
EOI-TF
Treatment Course
D
CR
TF
Diagnosis
Remission
Frequency CD4 TIM-3
Q1 6.97
Q2 1.37
Q4 49.4
Q3 42.2
Q1 5.57
Q2 2.89
Q4 50.8
Q3 40.7
Frequency CD4 PD-1
Frequency CD8 TIM-3
Q1 1.56
Q2 0.47
Q4 41.9
Q3 56.0
Q1 4.63
Q2 2.81
Q4 55.9
Q3 38.8
Frequency CD8 PD-1

Fig.1

The association between Gal9 and TIM-3 as prognostic marker for Selinexor+HiDAC Mito regimen.

At time of diagnosis, multi-parameter flow-cytometry was performed on bone marrow (BM) aspirates from 26 patients. A FITC (BioLegend Clone 581) conjugated anti-CD34 antibody was used to analyze frequencies of CD34+ AML cells and the remaining CD34- cell populations. (A). Patients were divided into 2 cohorts - those who achieved CR (n=16), and those who experienced TF (n=10). The comparison in the frequency of CD4+ (PerCPCy 5.5 Biolegend Clone SK3) PD-1+ (Pe Biolegend Clone EH12.2H7) T cells and CD34-Gal-9+ (APC BioLegend Clone 9M1-3) in these two group are shown. (B-C) The Mann-Whitney U test was used to compare the 2 groups. Bars represent medians. $P < 0.05$ was considered statistically significant. Percentage of cumulative frequencies are displayed in E and F. We calculated the Spearman correlation coefficients to describe associations between CD4+PD-1+ T cells and CD34+PDL-1+ (BV-421 BioLegend Clone 29E.2A3) AML cells in TF patients. ($r_s=1; p<0.0004$) and Gal-9 and inhibitory and activator markers on CD8+ (FITC BioLegend Clone HIT8a) cells at time of diagnosis (TIM-3 (APC Cy-7 BioLegend clone F38-2E2), ICOS (APC-Cy7), Lag3 (Pe-Cy7)). A linear regression according to the Deming procedure and deviation for linearity (Runs Test) was additionally computed. Runs Test was not significant. Gal-9 and TIM-3 vs HLA-DR (APC) was used as negative control. (I)

Fig.2

The increase of TIM-3 expression was higher in TF compare to CR patients

Median Fluorescence Intensity (MFI) was calculated by FlowJo-10 software and relative normalized to comparing TIM-3 expressing T cells (CD4+ and CD8+) and PDL-1+ CD34+ cells in CR and TF patients at time of diagnosis and end of induction (A-B). The Mann-Whitney test was used to compare the 2 sub-groups. Bars represent medians. $P < 0.05$ was considered statistically significant. The colored overlay dot plots shows the co-expression of TIM-3 and PD-1 on $CD4^+$ and $CD8^+$ cells comparing expression levels of these receptors in representative patients CR (blue dot) and TF (red dot) at diagnosis vs end of induction.

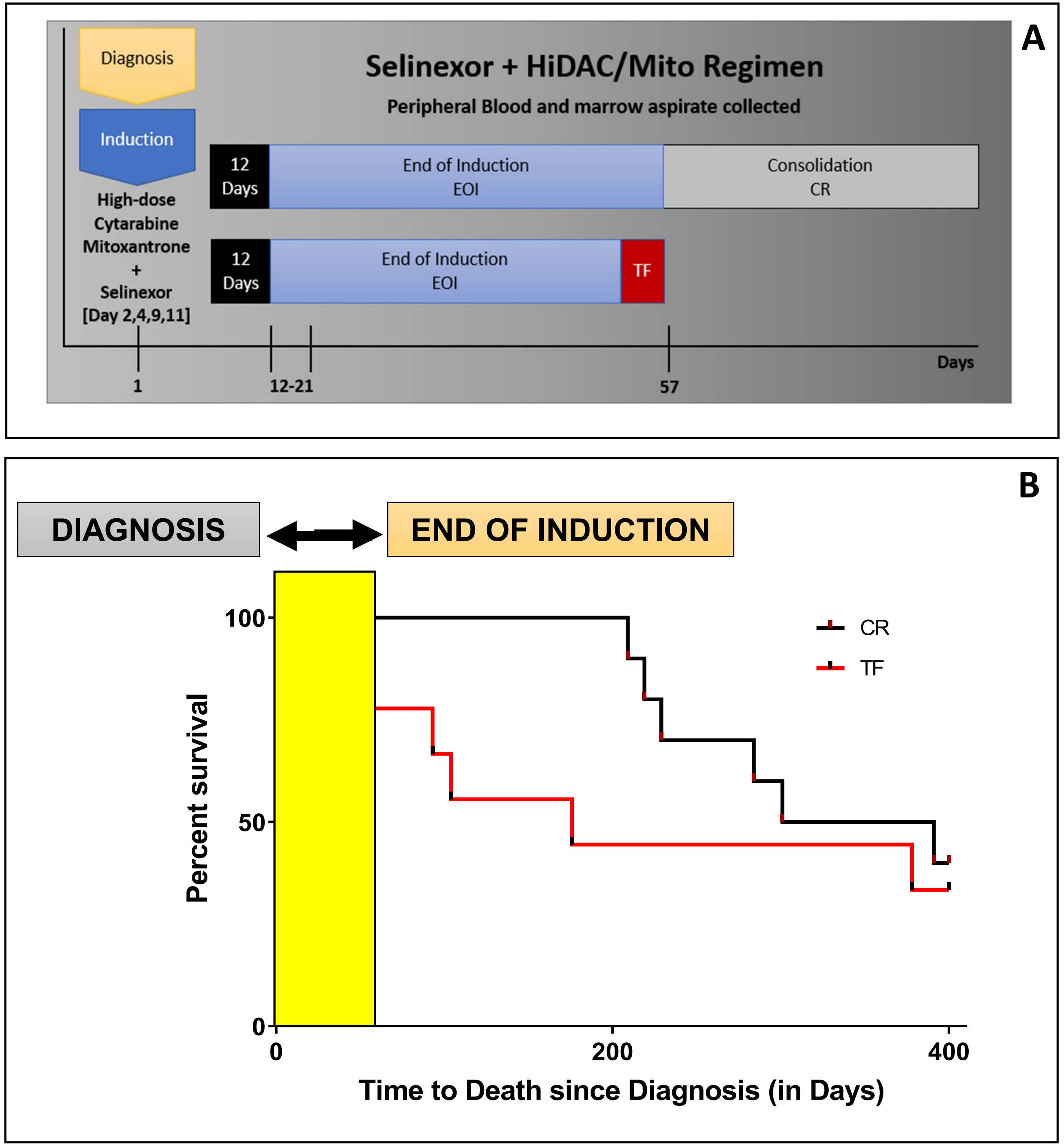

Supplementary Fig.1-A

Strategy of the previous study and samples collection.
26 patients divided in two group enrolled to a phase I dose escalation trial that combined increasing doses of Selinexor (SINE) with age-adjusted HiDAC/Mito (NCT02573363) at time of diagnosis. Patients experienced induction failure was taken off of protocol therapy due to death or documented induction failure. HiDAC (3 g/m2,or 2 g/m2 if > 70 years, intravenously over 4 h) followed immediately by Mito (30 mg/m2, or 20 mg/m2 if > 70 years, intravenously over 1 h) were administered on days 1 and 5. Selinexor was given orally on days 2, 4, 9, and 11. Initial Selinexor dose was 60 mg (~ 35 mg/m2 for an average adult) followed by dose escalation to a target level of 80 mg (~ 50 mg/m2). Bone Marrow (BM) and blood samples were collected at the time of diagnosis and at the end of induction/treatment (days range 19-56).

Supplementary Fig.1-B

Kaplan-Meier Plot curves depicting CR (black) and TF (red) patients survival (percent) since the time of the diagnosis. ~~The median of the days elapsed since the diagnosis was 346 and 176 days for CR and TF respectively and Hazard Ratio (Mantel-Haenszel) TF/CR was 1.7~~, Mantel-Cox test was used to compare the 2 groups. The shadow in the chart indicates the timewise of samples collection and analysis.

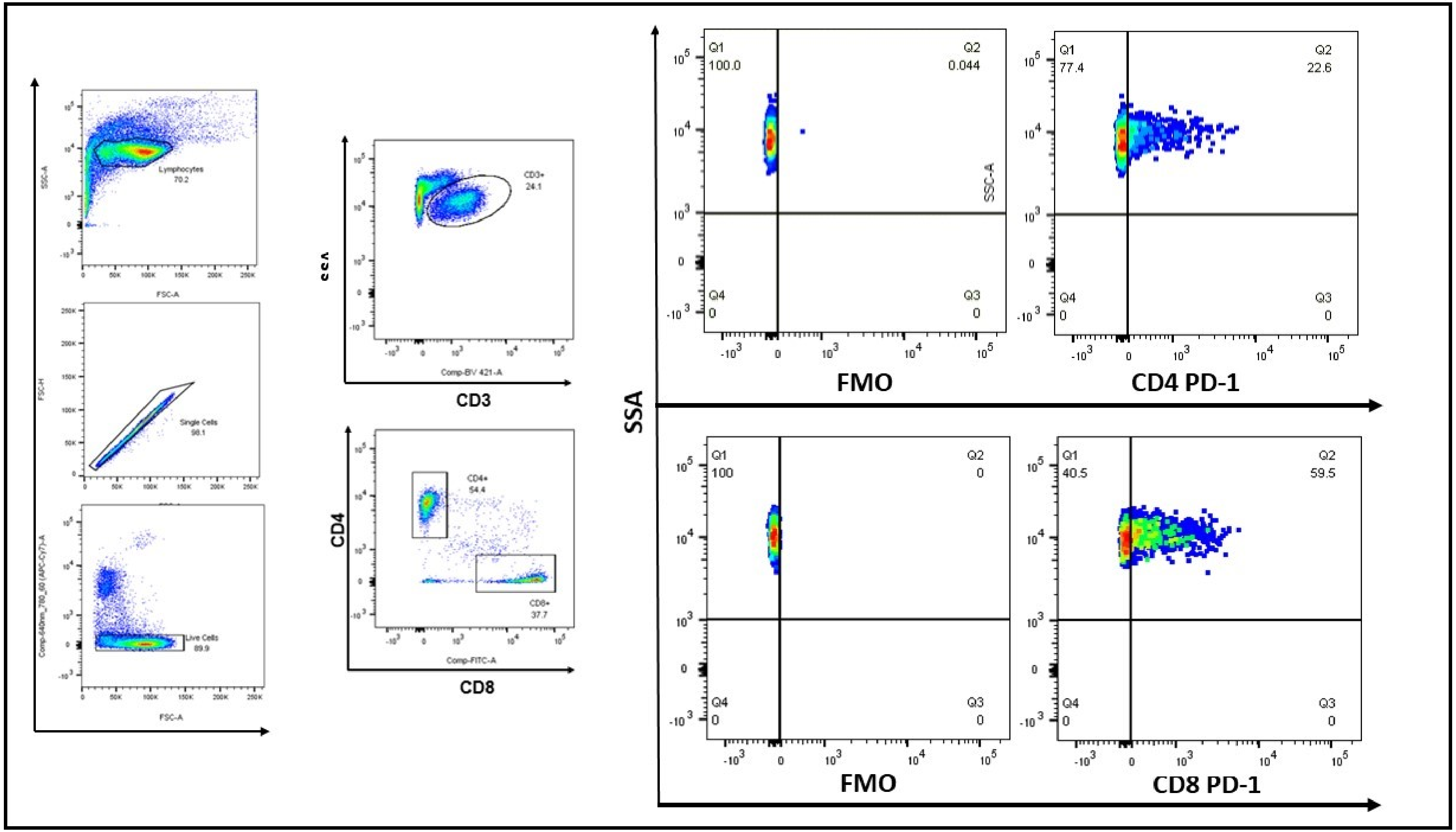

Supplementary Fig.2

Sequential gating to identify PD-1. Multi-parameter flow-cytometry was performed on blood and bone marrow (BM) aspirates. PD-1 was stained with Pe (BioLegend Clone EH12.2H7). A Fluorescence Minus One (FMO) controls were used to determine the median fluorescence intensity (MFI) and frequency among the parent population of each costimulatory and coinhibitory molecule. Here we show the frequency of PD-1 gated on CD4 and CD8 population

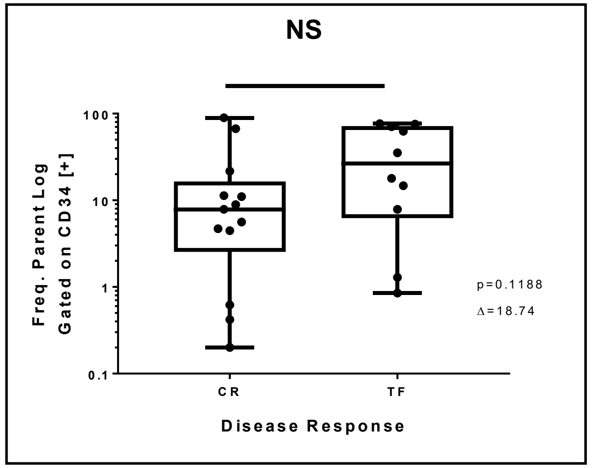

Supplementary Fig.3

Statistical trend toward higher frequencies of $CD34^+$ cells in TF patients

$CD34^+$ cells were stained with FITC (BioLegend Clone 541). Here is shown the frequency (Log scale) in comparison between TF and CR cohorts at the time of diagnosis. ~~The Mann-Whitney U test was used to compare the 2 groups. Bars represent medians. P<0.05 was considered statistically significant.~~

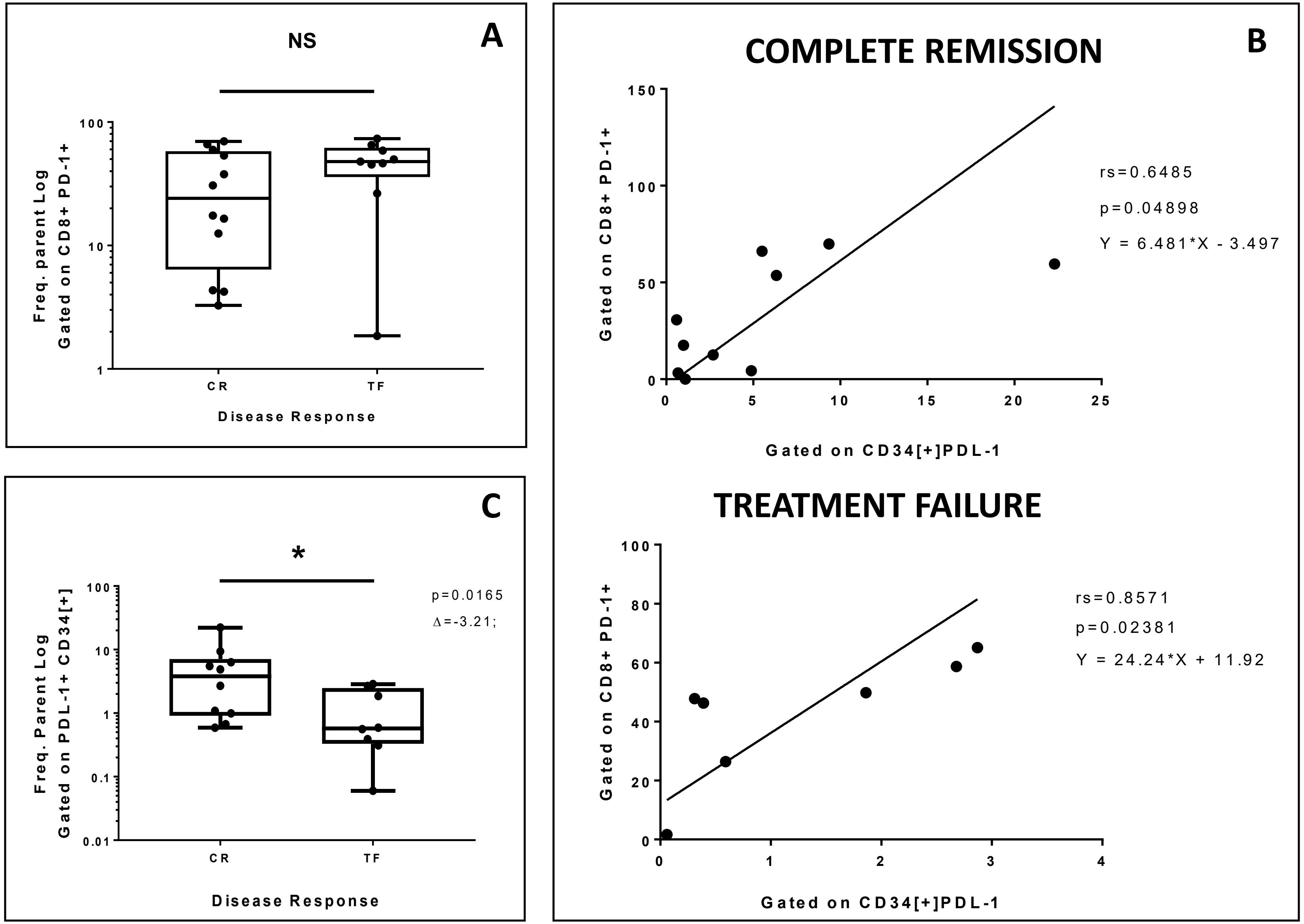

Supplementary Fig.4
PD-1/PDL-1 axes at the time of diagnosis as a prognostic factor.

In (A) is shown the frequency (Log scale) of $CD8^+$ ~~(FITC BioLegend Clone HIT8a)~~ $PD\text{-}1^+$ ~~(Pe Biolegend Clone EH12.2H7)~~ cells in comparison of TF and CR cohorts at the time of diagnosis. ~~The Mann-Whitney U test was used to compare the 2 groups. Bars represent medians. P < 0.05 was considered statistically significant.~~ (B) Spearman correlation coefficients in TF (above) and CR (bottom) populations between $CD8^+$ $PD\text{-}1^+$ cells and $CD34^+$ ~~(FITC BioLegend Clone 541)~~ $PDL\text{-}1^+$ ~~(BV-421 BioLegend Clone 29E.2A3)~~ cells. According to the Deming procedure linear regression equation is shown. ~~p<0.05 is considered statically significant.~~ In (D) Frequency (Log scale) $CD34^+$ $PDL\text{-}1^+$ cells in comparison of TF and CR cohorts at the time of diagnosis. ~~The Mann-Whitney U test was used to compare the 2 groups. Bars represent medians. P < 0.05 was considered statistically significant.~~

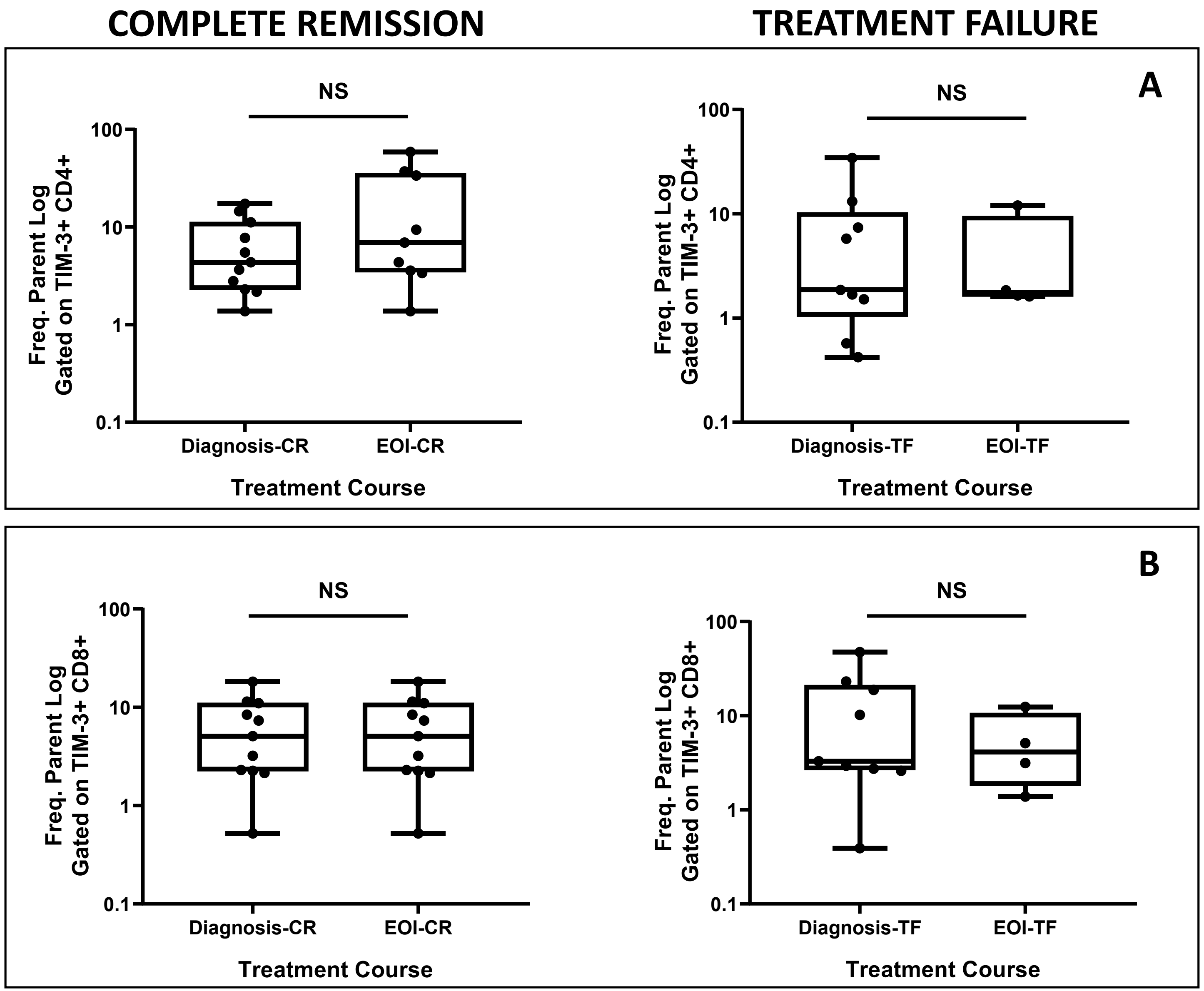

Supplementary Fig. 5
TIM-3 expression in TF and CR patients at diagnosis and at the end of induction.

In (A) is shown the frequency (Log scale) of CD4$^+$ ~~(PerCPCy 5.5 Biolegend Clone SK3)~~ TIM-3$^+$ ~~(APC Cy-7 BioLegend clone F38-2E2)~~ cells at the time of diagnosis and at the end of remission in CR (left) and TF (right) patients. In (B) is shown the frequency (Log scale) of CD8$^+$ ~~(FITC BioLegend Clone HIT8a)~~ TIM-3$^+$ cells. ~~The Mann-Whitney U test was used to compare the 2 groups. Bars represent medians. P<0.05 was considered statistically significant.~~

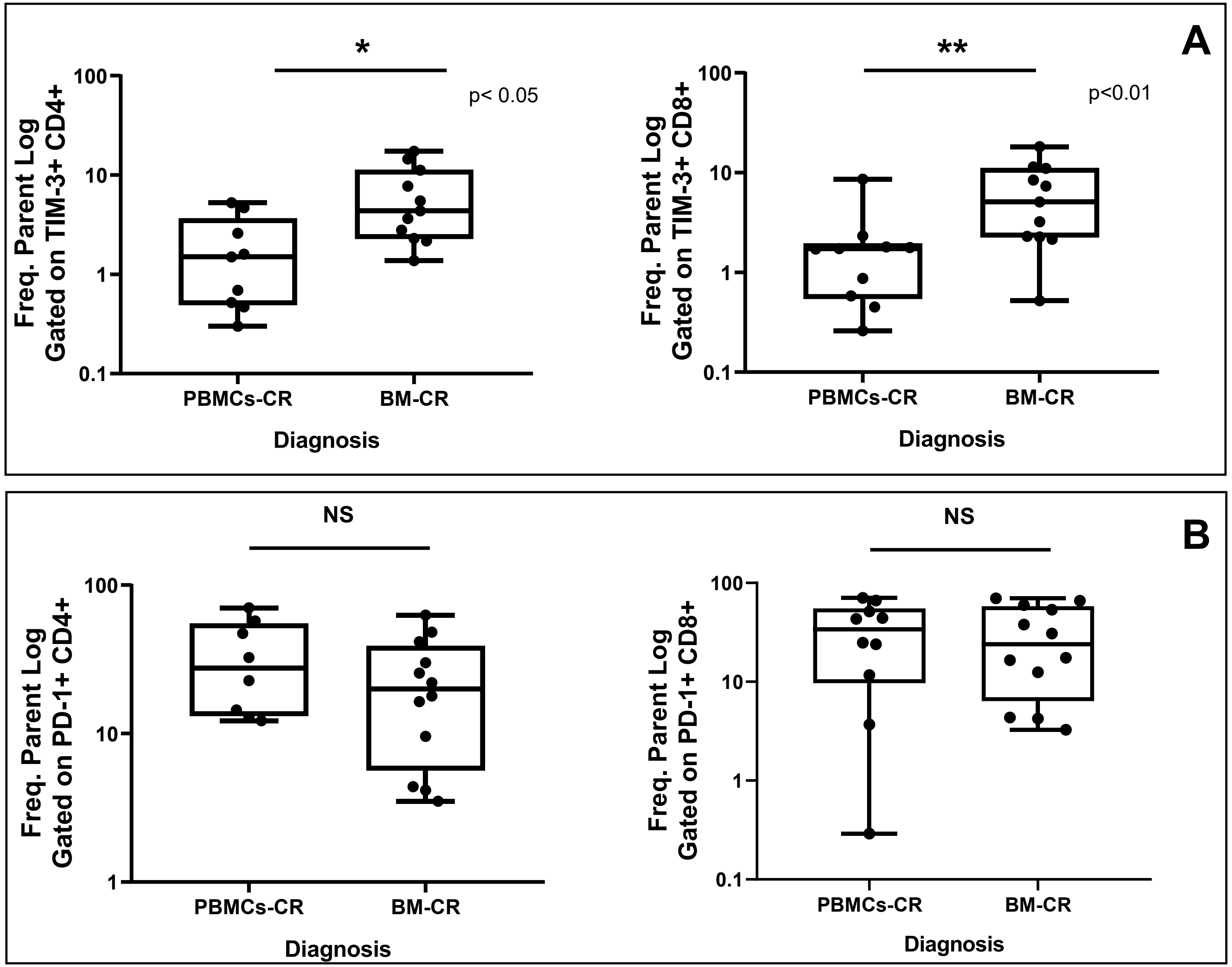

Supplementary Fig. 6
TIM-3 expression in BM compartment is significant higher in the both subsets of CD4 and CD8 populations

In (A) is shown the frequency (Log scale) of $CD4^+$ ~~(PerCPCy5.5 BioLegend Clone SK3)~~ $TIM\text{-}3^+$ ~~(APC Cy-7 BioLegend clone F38-2E2)~~ cells (right) and $CD8^+$ ~~(FITC BioLegend Clone HIT8a)~~ (left) at the time of diagnosis in comparison between peripheral blood and bone marrow compartments. ~~p<0.05 was considered statistically significant.~~
In (B) is shown the same comparison in the frequency (Log scale) of $CD8^+$ ~~(FITC BioLegend Clone HIT8a)~~ $PD\text{-}1^+$ ~~(Pe BioLegend Clone EH12.2H7)~~ cells. ~~The Mann-Whitney U test was used to compare the 2 groups. Bars represent medians. P<0.05 was considered statistically significant.~~

Gal9 EXPRESSION

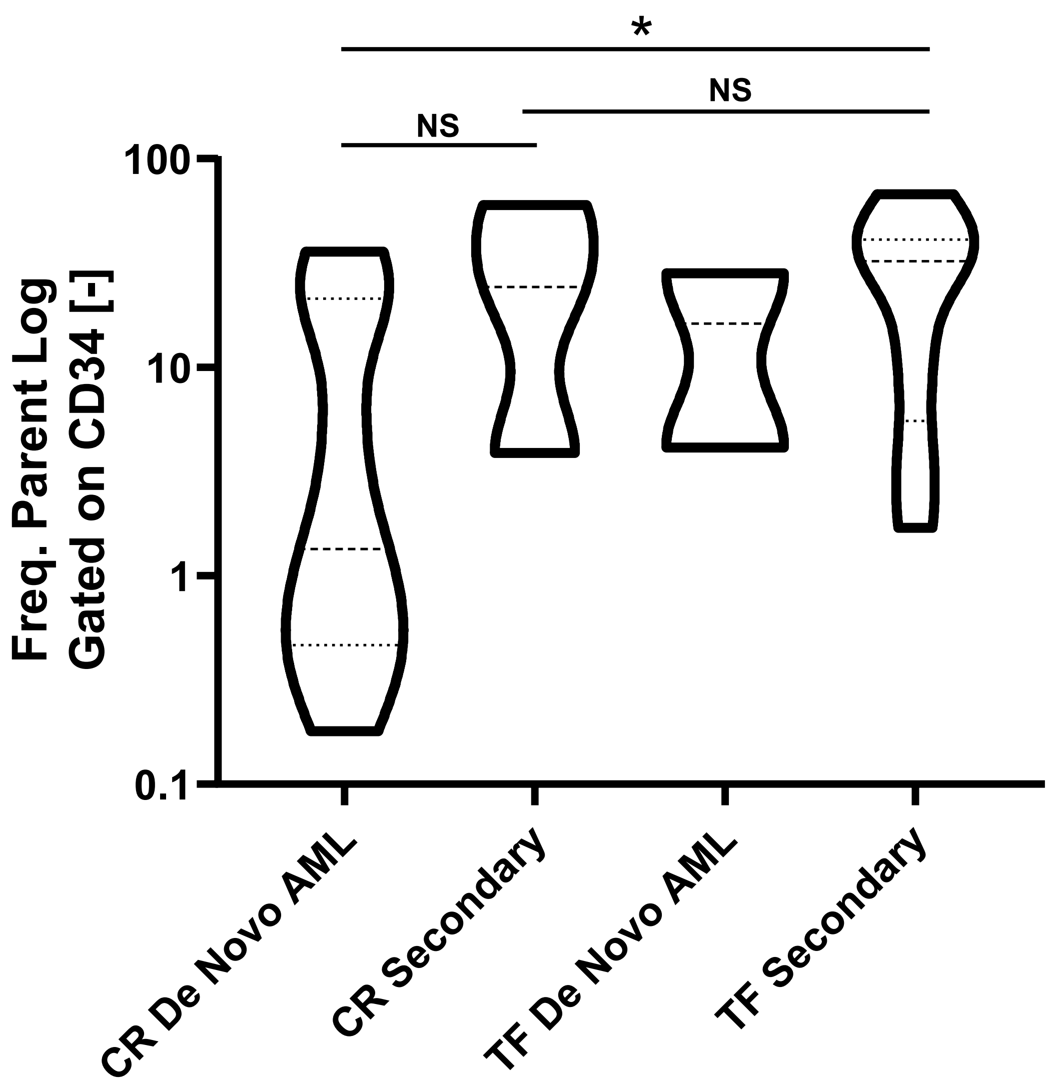

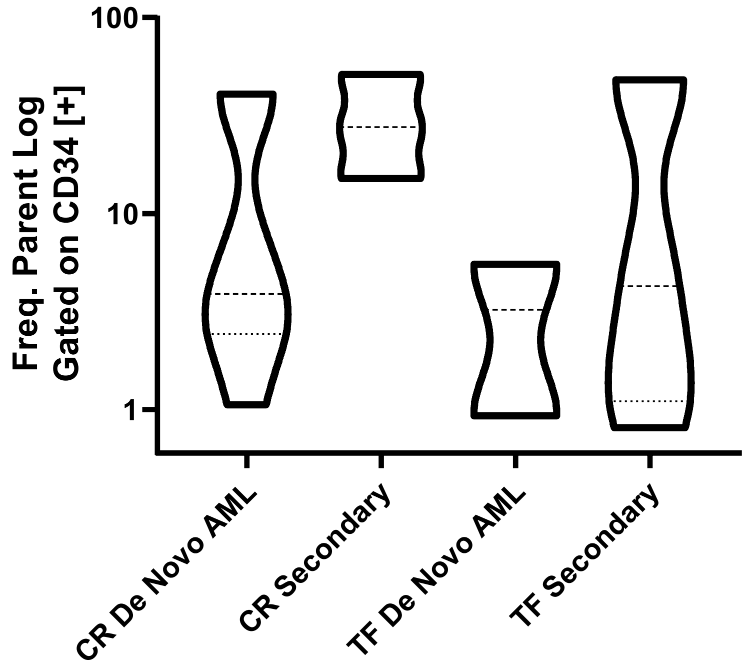

CD34+ EXPRESSION

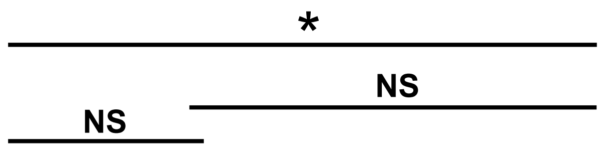

Table 1. Characteristic of the patients

	Disease Response	
	Complete Remission	Treatment Failure
Patient Characteristics	Number (%)	Number (%)
Total patients enrolled	16	10
Female	6 (37%)	8 (80%)
Median Age (years, range)	61 (35-75)	62 (38-74)
Disease State on enrollement*		
Untreated AML	12 (80%)	2 (22%)
Relapse or refractory AML	3 (20%)	7 (78%)
Initial AML diagnosis*		
De Novo AML	8 (53%)	5 (55%)
Secondary AML after MDS	7 (47%)	4 (45%)
Acquired Mutation Status*		
FLT3	3 (20%) NMP1 mutated	2 (22%) NMP1 mutated
CEPBA	1 (6%)	2 (22%)
NMP1	5 (34%)	1 (11%)
Blast (CD34+)		
Median	9.90%	48.90%
Range	4.5-89.7	7.9-76.6

* CR pts=15 TF* pts=9

Fig.1

The association between Gal9 and TIM-3 as prognostic marker for Selinexor+HiDAC Mito regimen.

At time of diagnosis, multi-parameter flow-cytometry was performed on bone marrow (BM) aspirates from 26 patients. A FITC conjugated anti-CD34 antibody was used to analyze frequencies of CD34+ AML cells and the remaining CD34- cell populations. (A). Patients were divided into 2 cohorts - those who achieved CR (n=16), and those who experienced TF (n=10). The comparison in the frequency of CD4+ (PerCPCy 5.5 Biolegend Clone SK3) PD-1+ (Pe Biolegend Clone EH12.2H7) T cells and CD34-Gal-9+ (APC BioLegend Clone 9M1-3) in these two group are shown. (B-C) The Mann-Whitney U test was used to compare the 2 groups. Bars represent medians. $P < 0.05$ was considered statistically significant. Percentage of cumulative frequencies are displayed in E and F. We calculated the Spearman correlation coefficients to describe associations between CD4+PD-1+ T cells and CD34+PDL-1+ (BV-421 BioLegend Clone 29E.2A3) AML cells in TF patients. ($r_s=1; p<0.0004$) and Gal-9 and inhibitory and activator markers on CD8+ (FITC BioLegend Clone HIT8a) cells at time of diagnosis (TIM-3 (APC Cy-7), ICOS (APC-Cy7), Lag3 (Pe-Cy7)). A linear regression according to the Deming procedure and deviation for linearity (Runs Test) was additionally computed. Runs Test was not significant. Gal-9 and TIM-3 vs HLA-DR (APC) was used as negative control. (I)

Fig.2

The increase of TIM-3 expression was higher in TF compare to CR patients

Median Fluorescence Intensity (MFI) was calculated by FlowJo-10 software and relative normalized to comparing TIM-3 expressing T cells (CD4+ and CD8+) and PDL-1+ CD34+ cells in CR and TF patients at time of diagnosis and end of induction (A-B). The Mann-Whitney test was used to compare the 2 sub-groups. Bars represent medians. $P < 0.05$ was considered statistically significant. The colored overlay dot plots shows the co-expression of TIM-3 and PD-1 on $CD4^+$ and $CD8^+$ cells comparing expression levels of these receptors in representative patients CR (blue dot) and TF (red dot) at diagnosis vs end of induction.

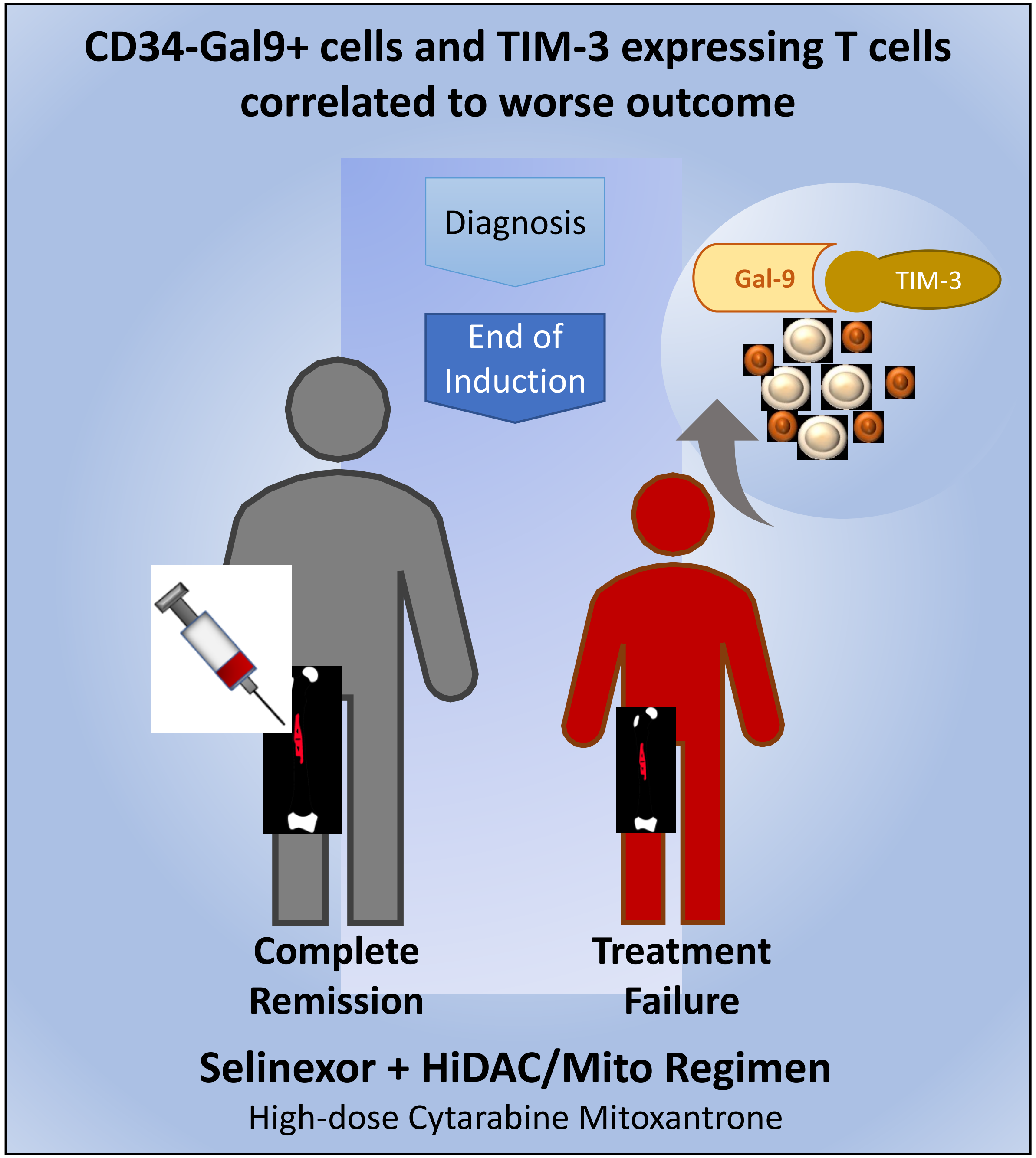
CD34-Gal9+ cells and TIM-3 expressing T cells correlated to worse outcome
Diagnosis
End of Induction
Gal-9
TIM-3
Complete Remission
Treatment Failure
Selinexor + HiDAC/Mito Regimen
High-dose Cytarabine Mitoxantrone
